# Carbon Mitigation Pathways of Urban Transportation under Cold Climatic Conditions

**DOI:** 10.3390/ijerph19084570

**Published:** 2022-04-11

**Authors:** Xianen Wang, Baoyang Qin, Hanning Wang, Xize Dong, Haiyan Duan

**Affiliations:** 1 College of New Energy and Environment, Jilin University, Changchun 130021, China; wxen@jlu.edu.cn (X.W.); qinby19@mails.jlu.edu.cn (B.Q.); 2 Key Laboratory of Groundwater Resources and Environment, College of New Energy and Environment, Jilin University, Ministry of Education, Changchun 130021, China; 3 Jilin Provincial Key Laboratory of Water Resources and Environment, School of New Energy and Environment, Jilin University, Changchun 130021, China; 4 Changchun Institute of Technology, Changchun 130012, China; wanghn@ccit.com

**Keywords:** transportation sector, cold regions, long-energy alternatives planning (LEAP) system, traffic ways, energy types, CO_2_ emission

## Abstract

Climate heterogeneity has enormous impacts on CO_2_ emissions of the transportation sector, especially in cold regions where the demand for in-car heating and anti-skid measures leads to high energy consumption, and the penetration rate of electric vehicles is low. It entails to propose targeted emission reduction measures in cold regions for peaking CO_2_ emissions as soon as possible. This paper constructs an integrated long-range energy alternatives planning system (LEAP) model that incorporates multi-transportation modes and multi-energy types to predict the CO_2_ emission trend of the urban transportation sector in a typical cold province of China. Five scenarios are set based on distinct level emission control for simulating the future trends during 2017–2050. The results indicate that the peak value is 704.7–742.1 thousand metric tons (TMT), and the peak time is 2023–2035. Energy-saving–low-carbon scenario (ELS) is the optimal scenario with the peak value of 716.6 TMT in 2028. Energy intensity plays a dominant role in increasing CO_2_ emissions of the urban transportation sector. Under ELS, CO_2_ emissions can be reduced by 68.66%, 6.56% and 1.38% through decreasing energy intensity, increasing the proportion of public transportation and reducing the proportion of fossil fuels, respectively. Simultaneously, this study provides practical reference for other cold regions to formulate CO_2_ reduction roadmaps.

## 1. Introduction

In recent years, with the rapid development in the economy, China has also made great progress in the transportation sector’s development [1]. The urban transportation sector, as the most significant part of the transportation sector, has the characteristics of large scale, high proportion and fast growth [2]. In 2015, the Paris Agreement put forward the goal of a global 1.5-degree temperature control, and a coordinated reduction in CO_2_ emissions is one of the approaches to achieving the goal [3]. In China, CO_2_ emissions of the transportation sector account for about 25% of China’s CO_2_ emissions, and CO_2_ emissions of the urban transportation sector accounts for about 40% of China’s transportation sector [4]. The urban transportation sector has become the third largest emitter following the industrial sector and the energy supply sector [5,6].

High energy consumption in the transportation sector leads to high CO_2_ emissions and consequently arouses interest in studying the driving factors of CO_2_ emissions from this sector. Raza et al. [7] quantified the impacts of carbon coefficient, fuel consumption, and total energy consumption on CO_2_ emissions from Pakistan’s transportation sector through the logarithmic mean Divisia index (LMDI). Li et al. [4] developed a National Energy Technology-Transport (NET-Transport) model to assess the impacts of shifting to alternative clean fuels, improving vehicle fuel efficiency and promoting public transportation on CO_2_ emissions from the urban transportation sector in China. Zhang et al. [8] and Zhang et al. [9], respectively, took Beijing and Yunnan as examples to explore the effects of the development of public transportation fuel structure on CO_2_ emissions of the urban transportation sector. In addition, other studies have analyzed the impact of fuel tax and fuel price subsidies on CO_2_ emissions from the transportation sector [1]. Among these factors, economic growth and fuel structure are the main contributors to increase CO_2_ emissions in the transportation sector. On the contrary, the energy intensity and the development of public transportation are the main contributors to reduce CO_2_ emissions, which have been confirmed in many other papers [6,10,11].

Based on the analysis of the driving factors of CO_2_ emissions, studies on the prediction of CO_2_ emissions in the transportation sector have also attracted extensive attention. In terms of methods of previous papers, there are mainly two types of models. Top-down models, such as the stochastic impacts by regression on population, affluence, and technology (STRIPAT) model [12] and the computable general equilibrium (CGE) model [13]; bottom-up models, such as the LEAP (long-energy alternatives planning) model [14] and NET-Transport (a sub-model of NET) [4]. Fang et al. [15] combined the STIRPAT model (top-down model) and scenario analysis to predict CO_2_ emission trajectories of 30 Chinese provinces, but the results are slightly discrepant from those calculated using bottom-up models. Top-down models cannot reveal the impact of policies on macroeconomy because all the macroeconomic and structural variables, such as economic growth rate and consumption, need to be determined externally [8]. Zhang et al. [9] analyzed the peak CO_2_ emission targets by applying the STIRPAT and LEAP models to data from Yunnan; the results of the LEAP model are regarded as more accurate because of detailed parameters and data. Given its advantages in alternative predictions, accuracy and policy settings, the LEAP model has been widely applied in predicting study at a national [16], regional [17] and sectoral scale [8].

China has a wide geographic area and a diverse terrain, forming various climates. China is divided into seven main climate zones according to the disparate from local climate conditions, including a cold region, chill region, hot summer and warm winter region, hot summer and cold winter region and mild region (Building Climate Regionalization Standard, 1994) [18]. Climate has a profound impact on human life, production and social activities, leading to significant regional CO_2_ emission disparities in different regions [19]. Previous papers have studied the impacts of climate on CO_2_ emissions of various sectors such as building [20,21], agriculture [22], electricity [23] and residential [24]. Using data from 30 provinces of China’s transportation sector, Liu et al. [25] evaluated the regional differences of carbon emission intensity (CEI). However, because of the difference in climate, traffic ways and passenger behavior, future trends of CO_2_ emissions in transportation sector varied significantly [26,27]. Typically, the cold region (January average temperature ≤ 10 °C, including Heilongjiang, Jilin, etc.) has a great difference between other regions in terms of traffic ways, fuel structure and public transportation share ratio due to the climate. When considering climatic heterogeneity, the probability of a biased conclusion is reduced in the prediction of CO_2_ emissions from the transportation sector in cold regions.

To the best of our knowledge, few studies have focused on the impacts of climate on CO_2_ emissions in the urban transportation sector. Especially in cold regions, the demand for in-vehicle heating and anti-skid measures leads to high energy consumption and low penetration of electric vehicles. It entails to propose targeted emission reduction measures in cold regions for peaking CO_2_ emissions. Additionally, from the experience of previous studies, the LEAP model is suitable for addressing the issues that this study is targeting.

This study establishes the LEAP model of the urban transportation sector for cold regions based on the data (2017) of urban traffic ways, fuel structure and public transportation proportion in cold regions extracted by the National Bureau of Statistics. We predict the CO_2_ emissions in the urban transportation sector and quantify the contributions of the driving factors to CO_2_ emission reduction in cold regions by using the LEAP model. Finally, this paper explores the targeted CO_2_ emission reduction measures for cold regions from the perspective of climatic heterogeneity and proposes CO_2_ reduction pathways for the urban transportation sector in cold regions. This paper is aimed to provide a practical reference value for other cold regions.

Section 2 introduces the methodology of CO_2_ accounting in the transportation sector in a cold climatic condition, scenario settings and data sources; Section 3 gives the detailed empirical results of the transportation sector and performs sensitivity analysis; Section 4 provides the discussion and policy implications of this study; the conclusions are drawn in Section 5.

## 2. Methods and Data

### 2.1. Research Framework

**Figure 1:**
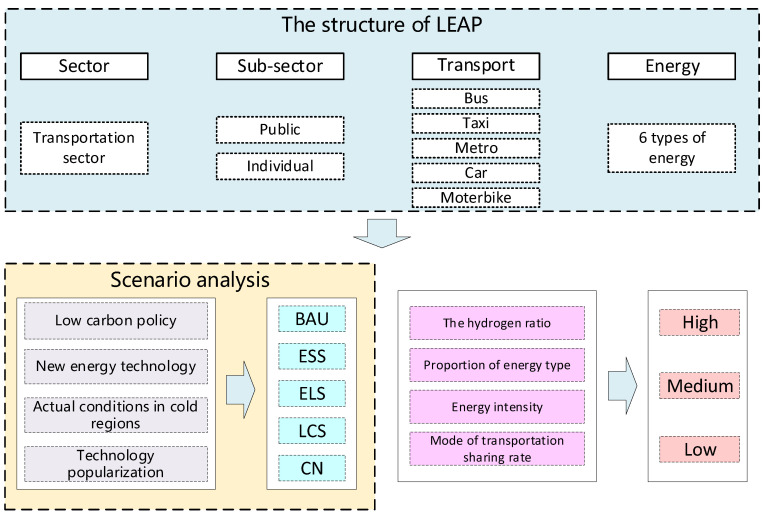
Research framework.

In order to aid the understanding of the methodological system, we present a schematic framework, as shown in Figure 1. The whole framework consists of three parts. First, the LEAP model of the transportation sector is built. According to the service provider, the urban transportation is divided into public transportation and individual transportation. The public transportation is further divided into bus, taxi and the metro, and the individual transportation is divided into private car and motorbike. Second, from a regional perspective, five different scenarios are set according to the severity of low-carbon policies, the development of new energy technologies and the popularization of technologies: Business-As-Usual Scenario (BAU), Energy-Saving Scenario (ESS), Energy-Saving–Low-Carbon Scenario (ELS), Low-Carbon Scenario (LCS) and Carbon Neutral Scenario (CN). Third, from the variation setting perspective, the CO_2_ emission trend of the urban transportation sector in different scenarios is predicted through three kinds of growth rates of high, middle and low. Finally, the driving factors in each scenario and CO_2_ emission reduction pathways in cold region are analyzed.

### 2.2. Long-Range Energy Alternatives Planning System (LEAP) Model Framework

**Figure 2:**
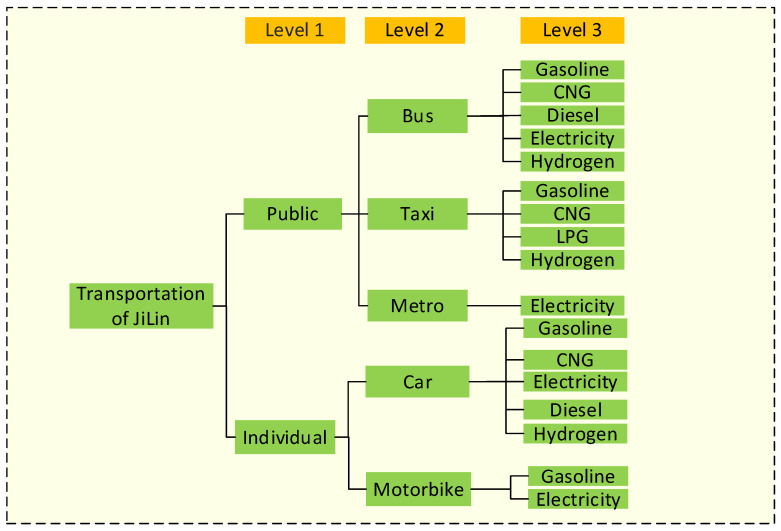
The structure of the LEAP model.

The LEAP model is an energy-planning system developed by the Stockholm Environment Institute and the University of Boston, and it is widely used to analyze energy policy and assess climate change mitigation [14]. LEAP is a prominent model that has been used in 190 countries, 85 UNFCCC country reports, and more than 70 peer-reviewed journal papers [16]. To address the issue of data availability, the LEAP model developed the Technology and Environmental Database (TED), which can be used as a stand-alone tool or as part of the LEAP model to calculate carbon emissions from different energy mitigation options. The LEAP model has the characteristics of transparent data input, high flexibility and can be flexibly adjusted according to the existing data. The LEAP model developed in this paper is in line with the specific situation of the urban transportation sector in cold regions. The LEAP model consists of three levels, with 2017 as the base year and 2060 as the final year. According to the relevant statistical data, level 1 consists of public transportation and individual transportation. Level 2 includes bus, taxi, the metro, car and motorbike. Since bicycles and walking do not use energy, they are not discussed in the model. In level 3, the energy resources mainly consist of gasoline, diesel, LPG, CNG, electricity and hydrogen. The scenario setting and parameter setting will be detailed in Section 2.5. The specific model structure is shown in Figure 2.

### 2.3. CO_2_ Emissions Accounting Method

The standardized methodology published by the IPCC Guidelines was used to estimate the CO_2_ emissions of the transport sector [8]. This approach has obvious advantages in calculating the CO_2_ emissions of China’s transportation sector. In summary, the method involves three parameters: energy type, energy consumption and carbon emission factor. The carbon emission factor for fossil fuels is obtained by multiplying the average low-order calorific value, average carbon content and carbon oxidation rate. The formula for calculating carbon emissions from fossil fuels is as follows:

$$C=\sum_{i}C_{i}=\sum_{i}FC_{i}\times F_{i}=\sum_{I}FC_{I}\times ALC_{i}\times c_{i}\times R_{i}\times \frac{44}{12}$$

where *C* (kg CO_2_) represents CO_2_ emissions generated by fossil energy consumption; *i* refers to the different types of fossil fuels (including petrol, diesel, LPG and natural gas); *FC* (kg) represents consumption; *F* (kg CO_2_/kg) is the carbon emission coefficient; *ALC* (kJ/kg) is the average low-order calorific value; *c* (T/TJ) is the carbon content and *R* is the oxidation rate of carbon.

### 2.4. Research Zone

With the Revitalizing Outdated Industrial Region of Northeast China strategy, northeast China has become a typical developing region with rapid industrialization and urbanization, high energy consumption and substantial CO_2_ emissions. Meanwhile, cities in northeast China play an important role in regional low-carbon developments. Jilin is a typical cold region located in northeast China. According to the Building Climate Regionalization Standard [18], the average temperature of Jilin in January is less than or equal to minus 10 degrees Celsius, which is classified as a cold region. Jilin has the typical traffic ways and energy types in cold regions. Economic development is relatively backward, while the automobile industry is relatively developed. Thus, we take Jilin as an example to study the path of CO_2_ emission reduction in the urban transportation sector. This study will provide a reference for CO_2_ emission reduction in the urban transportation sector in similar cold regions.

### 2.5. Scenarios and Data

This paper designed five scenarios for analyzing the CO_2_ emission trends resulting from the implementation of low-carbon policies of different intensities. According to the New Energy Vehicle Industry Development Plan (2021–2035) [28], electric vehicles will become the mainstream in the future, and the electric transformation of public transportation and the commercialization of fuel cells will be realized before 2035. However, because the endurance of new energy vehicles may be insufficient under cold climatic condition, the popularity of electric vehicles will be slightly affected. The scenario analysis timespan covers the years 2017–2060 with 2017 as the baseline year.

Parameters are set according to relevant contents in the Jilin Natural Gas Utilization Plan (2016–2025) [29]. Without considering the development of hydrogen energy in BAU, this study set the hydrogen energy ratio at 5.9% for ESS and ELS and 15.9% for LCS and CN. The energy consumption of public transportation in Jilin is mainly generated by buses and taxis. The main terminal energy types of buses are gasoline, CNG, diesel, electric power and hydrogen. Taxi terminal energy types are mainly gasoline, CNG, LPG, electricity and hydrogen energy. The energy consumption of individual transportation in Jilin is mainly generated by private cars and motorcycles. In cold regions, motorcycle travel in winter is not safe, and people prefer private cars to travel. Meanwhile, the use of snow tires and car heating will increase CO_2_ emissions. The main terminal energy types of private cars are gasoline, CNG, diesel, electric power and hydrogen. The terminal energy types of motorcycles are mainly gasoline and electricity. The parameter settings are shown in Table 1. The detailed descriptions for the five scenarios are given below:

**Table 1:** Parameter settings under different scenarios.

Scenarios	Parameter Descriptions	References
**BAU**	Energy efficiency and traffic ways are maintained at the current levels, without considering the development of hydrogen, energy intensity decreased by 0.1% in 2060.	Jilin Province Highway and Waterway Transportation Development “Thirteenth Five-Year Plan” [30]. The Outline of Transport Development Plan of Changchun during the 13th Five-Year Plan Period [31].
**ESS**	Improving the energy structure is the main objective of ESS. Natural gas and electricity ratio will account for 35.71% in 2030, 54.65% in 2050 and 80% in 2060. The proportion of hydrogen will be 5.9% in 2060, energy intensity will decrease by 0.1% in 2060.	China Renewable Energy Development Report 2020 [32]. 2020 Changchun Urban Transport Development White Paper [33]. Research on the Innovative Development of Hydrogen Energy Industry in Jilin Province under the Background of New Infrastructure Construction [34].
**ELS**	New energy vehicles will become a mainstream product, the proportion of new energy will account for 60% in 2030, 75.36% in 2050, and basically realizing electric transformation in 2060. The proportion of hydrogen will be 5.9%, energy intensity will decrease by 0.2% in 2060.	Natural Gas Utilization Plan of Jilin Province (2016–2025) [29]. Report on China’s carbon peak by 2030 [35]. Research on the Innovative Development of Hydrogen Energy Industry in Jilin Province under the Background of New Infrastructure Construction [34].
**LCS**	The development of public transportation is a major goal of LCS. Public transportation will account for 35% in 2030, 58.63% in 2050, and 70% in 2060. The proportion of bus use will increase to more than 80% in 2060. Public transportation will be completely electrified in 2060. Energy intensity will decrease by 0.2% in 2060.	New Energy Vehicle Industry Development Plan (2021–2035) [28]. Hydrogen Energy Industry in Jilin Province under the Background of New Infrastructure Construction [34].
**CN**	The proportion of gasoline and diesel in the terminal energy will be reset by 2060, electricity and hydrogen will account for 80% and 15.9%, respectively, in 2060. Energy intensity will decrease by 1% in 2060.	Research Report on China’s Carbon Neutrality by 2060 [36].

#### 2.5.1. Business-As-Usual Scenario (BAU)

In BAU, we assume that energy efficiency and traffic ways are maintained at the current levels. Meanwhile, further mitigation and energy saving policies or planning are not taken into consideration. Under cold conditions, people prefer taxis and the metro because they are not willing to wait for a bus. Therefore, in the absence of better measures, the proportion of bus use is relatively small. In 2060, clean energy maintains its dominance, comprising more than 60%, without considering the development of hydrogen in BAU. The energy intensity decreased by 0.1% in 2060.

#### 2.5.2. Energy-Saving Scenario (ESS)

Improving the energy structure is a major goal of ESS. In ESS, we assume that gasoline and diesel for all vehicles will be substituted by natural gas and electricity with the percentage share of reaching from 50 % to 80% by 2060. In 2060, clean energy maintains its dominance, comprising more than 65% and hydrogen accounts for 5.9%. The energy intensity decreased by 0.1%. In summary, the ESS shows the development and emission status under the energy-saving policy in cold regions.

#### 2.5.3. Energy-Saving–Low-Carbon Scenario (ELS)

In ELS, the harmonization of the economy increases, and emission reduction is recognized as a key issue in the context of a low-carbon society. The energy intensity will be reduced by 0.2%, the metro and the use of electricity will be vigorously developed. Above all, hydrogen is beginning to be used in public transportation, and hydrogen accounts for 5.9% in energy structure. This scenario simulates the coordination between the economy development and emission reductions, more emphasizing energy efficiency and the application of low-carbon technologies, such as new energy vehicle technology and transportation equipment exhaust gas purification technology.

#### 2.5.4. Low-Carbon Scenario (LCS)

The development of public transportation is a major goal of LCS. The LCS is a relatively ideal solution. Technological improvements will accelerate the phasing out of high-energy and heavily polluting petrol and diesel vehicles. Gasoline and diesel will account for less than 10% of the total terminal energy and clean energy maintains its dominance, comprising more than 70%, and hydrogen accounts for 15.9%. This scenario shows the maximum reductions that can be achieved in order to balance the economy and environment.

#### 2.5.5. Carbon Neutral Scenario (CN)

Under CN, CO_2_ emissions will be cut tempestuously, since vehicles with high energy consumption and heavy pollution will be phased out after 2030. Meanwhile, the proportion of gasoline and diesel in the terminal energy will be reset by 2060. Energy intensity will be reduced by 1%, gasoline and diesel will be completely replaced, and electricity and hydrogen will account for 80% and 15.9%, respectively. The CN represents the optimal level of technological development, and the problem of fuel cell endurance at low temperatures will be solved.

The data of energy consumption by energy sources derive from national statistical yearbooks [37] and the Jilin Statistical Yearbook [38], the data of the proportion of traffic ways came from the China Transportation Statistical Yearbook [39], and the CO_2_ emission was calculated by using the coefficient of carbon dioxide emission by the Intergovernmental Panel on Climate Change (IPCC) Guidelines [40].

## 3. Results

### 3.1. Peak Time and Peak Value in the Urban Transportation Sector

**Figure 3:**
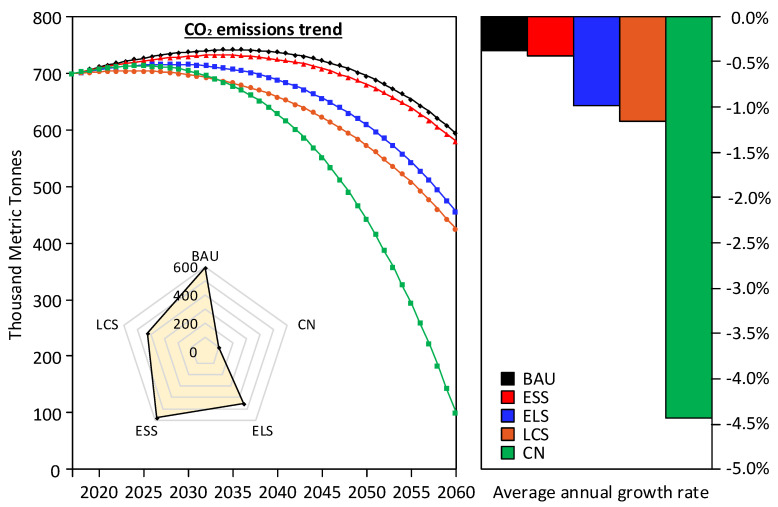
CO_2_ emissions trend of the urban transportation sector in Jilin. The line chart (**left**) shows the trends in CO_2_ emissions from 2017 to 2060 under five scenarios for the transport sector. The bar chart (**right**) shows the average annual growth rate of CO_2_ emissions from the transport sector under different scenarios. The radar map (**left**) shows the state of CO_2_ emissions in the transport sector under different scenarios in 2060.

As shown in Figure 3, LEAP is used to predict the CO_2_ emissions of the transportation sector in Jilin under five scenarios. The results indicate that the peak values under the five scenarios are between 704.7 TMt and 742.1 TMt. Peak year of BAU is 2035, and the peak value is 742.1 TMt. The peak value of ESS is 732.4 TMt in 2033. In ELS, the peak year is 2028, and the peak is 716.6 TMt. The peak year under LCS is 2025, and the peak is 713.9 TMt. The peak year under CN is 2023, and the peak value is 704.7 TMt. With the exception of BAU and ESS, the other three scenarios all reach the peak value of CO_2_ emission before 2030, and the peak time gradually moves forward with the degree of policy stringency. During 2017–2060, the average annual growth rate of CO_2_ emissions under the five scenarios is −0.38%, −0.44%, −0.99%, −1.15% and −4.43%, respectively. Although the peak time and peak value under CN are the best in the five scenarios, this scenario is not realistic for mid-to-late industrialization and urbanizing developing regions, such as Jilin. Limiting social and economic development to reduce CO_2_ emissions, the LCS can no longer meet the requirements of improving living standards. To sum up, it is impossible for Jilin to achieve a net zero emission under CN, and an ideal low-carbon society under LCS in the short-term also ignores future energy saving policies and new technologies. Therefore, ELS is the suitable situation of Jilin.

### 3.2. CO_2_ Emissions of Different Traffic Ways and Energy Types

**Figure 4:**
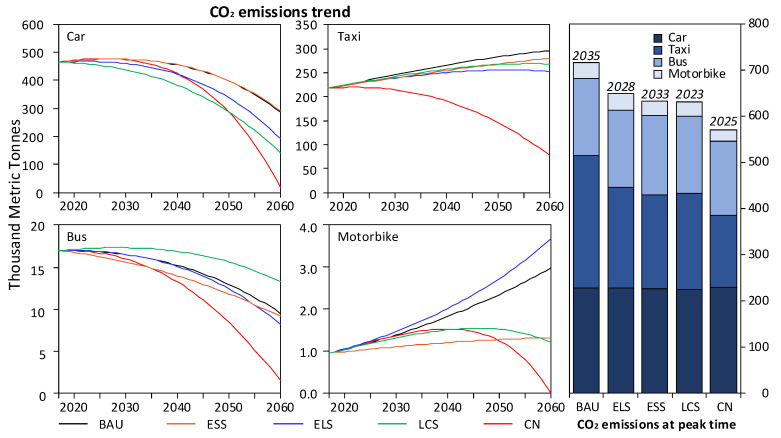
CO_2_ emissions of different traffic ways at peak time in transportation sector of Jilin. The line chart (**left**) is the CO_2_ emission trend of different traffic ways under different scenarios from 2017 to 2060. The bar chart (**right**) shows CO_2_ emissions of different traffic ways under different scenarios in peak time.

Due to the low share of motorbikes under cold climatic conditions, motorbikes are not considered as the key research objects, and the research mainly focuses on private cars, taxis and buses. In general, cars in cold regions account for the largest proportion of CO_2_ emissions in the transportation sector, followed by taxis and buses. People in cold regions are more inclined to buy private cars due to the low temperature, difficulties in taking a taxi and long waiting time for buses. As shown in Figure 4, the CO_2_ emission generated by car ranges from 224.6 TMt to 230.2 TMt, accounting for 24.4% to 35.9% of the total CO_2_ emissions. Car heating in winter increases the CO_2_ emissions in cold regions, and people prefer private cars to travel because of the cold climate. The CO_2_ emissions generated by taxis are 159.8–170 TMt, accounting for 23% of the total CO_2_ emissions. The cold climate also increases the probability of people taking taxis, especially in extreme climates, rainy and snowy days. The CO_2_ emission of buses is between 57 TMt and 87.9 TMt, accounting for 8–12% of the total CO_2_ emissions. The CO_2_ emission of motorbikes is less, in the range of 24.4–35.9 TMt, and their share in the total CO_2_ emissions is basically stable, at about 5%. In addition, the use of snow tires on all types of vehicles also increases the CO_2_ emission. Under ELS, cars, taxis, buses and motorbikes emit 226.3, 164.4, 82.4 and 35.9 TMt in peak years, respectively, accounting for 32%, 23%, 11% and 5% of the total CO_2_ emissions. Other activities account for a relatively small proportion, and the metro and hydrogen-powered vehicles do not directly contribute to CO_2_ emissions. As a result, cars, taxis and buses account for more than 60% of CO_2_ emissions in the transport sector in cold regions. On this basis, it is believed that these three ways of traffic are the key influencing factors to promote CO_2_ emission reduction.

According to Figure 4, the peak of CO_2_ emissions of cars in Jilin under BAU, ELS, ESS, LCS and CN scenarios are 227.8 TMt, 228.8 TMt, 226.3 TMt, 224.6 TMt and 230.2 TMt, respectively. The share of fossil energy in the peak period under BAU is 27.46%, and the proportion of fossil energy fuel in the other four scenarios are 33.13%, 22.45%, 22.57% and 0%, respectively. In the cold regions, more people tend to use conventional fuel vehicles because fuel cell vehicles are difficult to start in a short time and a long warm-up time is required in low temperature conditions.

**Figure 5:**
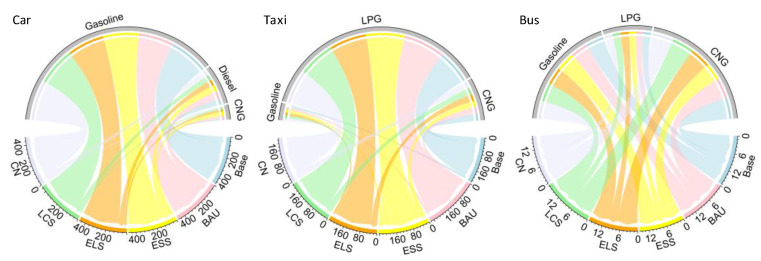
CO_2_ emissions of different traffic ways in base year and peak time (BAU, ESS, ELS, LCS, CN) in transportation sector of Jilin (unit: TMt). For example, in the chord diagram (**left**), carbon emissions of cars in the CN scenario are 404.92 tons from gasoline consumption, 57.74 tons from diesel consumption and 14.42 tons from CNG consumption in peak time.

For cars, the comparison of the five scenarios reveals that the energy structure has a significant effect on the CO_2_ emission of cars. Since hydrogen is introduced into ELS and LCS, fossil energy accounts for a relatively large proportion in these two scenarios, but the total CO_2_ emissions are not tremendous. The results prove that the optimization of the energy structure can decrease the CO_2_ emissions. In addition, slowing down the growth of urbanization, limiting the increase in private cars, promoting energy-saving technologies and improving energy efficiency can effectively reduce the CO_2_ emissions produced by cars. As for taxis, Figure 5 shows that under BAU, ELS, ESS, LCS and CN, the peak of CO_2_ emissions of taxis in Jilin are 170 TMt, 166.7 TMt, 164.4 TMt, 162.7 TMt and 159.8 TMt, respectively. The comparison of the five scenarios indicates that there is little difference in the share of terminal energy fuel of the five scenarios. As Jilin vigorously promotes the change of taxis from oil to gas during the 13th Five-Year Plan period, the energy structure has no particularly significant impact on the CO_2_ emissions of taxis. However, an important source of CO_2_ emissions from taxis is internal heating in winter. Therefore, improving energy efficiency, reducing empty-loading ratio and strengthening road transportation infrastructure construction can effectively reduce CO_2_ emissions from taxis. As for buses, it can be seen from Figure 5 that under BAU, ELS, ESS, LCS and CN, the peak of CO_2_ emission of buses in Jilin is 15.97 TMt, 15.19 TMt, 16.68 TMt, 17.25 TMt and 16.66 TMt, respectively, and the proportion of electricity and hydrogen is 48%, 40.7%, 39.2%, 32.7% and 39.4%, separately. The comparison of the five scenarios proves that increasing the proportion of clean fuel has an obvious stimulative effect in CO_2_ emission reduction. Therefore, it is necessary to continue to increase the pace of bus electrification, and basically realize the electric transformation before the peak of CO_2_ emissions.

### 3.3. The Reduction Pathway of CO_2_ Emissions

**Figure 6:**
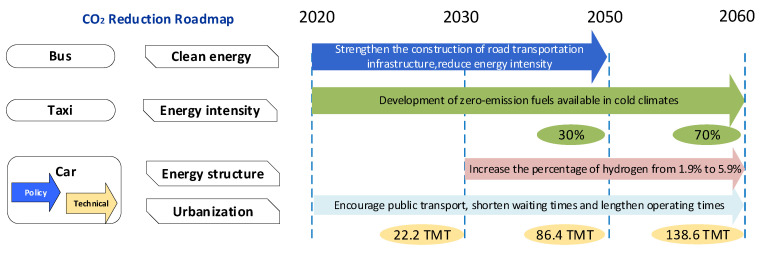
CO_2_ reduction roadmap for the urban transportation sector in ELS.

According to the ELS, new energy vehicles will become a mainstream product, basically realizing electric transformation in 2060. Reducing coal and oil consumption would increase the proportion of electricity to 30% by 2030 and 70% by 2060, separately. As shown in Figure 6, by 2030 and 2060, the share of hydrogen in terminal energy will increase to 1.9% and 5.9%, respectively. By 2060, the terminal energy intensity of each branch will be reduced by 0.2%, and the metro transit will account for more than 40% of the total CO_2_ emissions. In the short term, the construction of public transport infrastructure would be strengthened in cold regions to promote the implementation of the public transportation priority policy, such as shortening the waiting time, lengthening the running time of vehicles, reducing the empty load rate and enhancing energy efficiency. In the long run, in order to decrease the growth of CO_2_ emissions, zero-emission fuels available in cold regions would be developed; the durability of fuel cells at low temperatures would be promoted; the utilization of hydrogen would be expanded; the research and development of hybrid combustion technology of hydrogen and natural gas would be accelerated; and the construction density and the amount of charging piles would be increased. Before 2030, electric vehicles are difficult to widely popularize in cold regions, and the peak time cannot be greatly advanced. After 2030, if technological breakthroughs accelerate the adoption of electric cars, the peak time could be moved much earlier. In the long term, actions toward clean-energy vehicles, and the development of public transportation should receive more attention based on the mitigation-effect calculations of the LEAP model.

As for ESS, due to technological advances, natural gas has been used widely in the field of public transportation. However, road transportation infrastructure, especially gas stations and charging piles, should be strengthened in cold regions. The peak time under LCS is 2025. From the policy point of view, the population growth is slowing down, and a low-carbon society is promoted. The level of urbanization is significantly lower than the other three scenarios. Taxis and buses that run on gas and electricity will rapidly spread. In CN, it is difficult to achieve zero emissions in the transportation emissions generation stage without fully replacing terminal fuels with electricity. Due to the development of the hydrogen industry, the proportion of hydrogen in terminal energy will reach 15.9% by 2060.

### 3.4. Sensitivity Analysis

**Figure 7:**
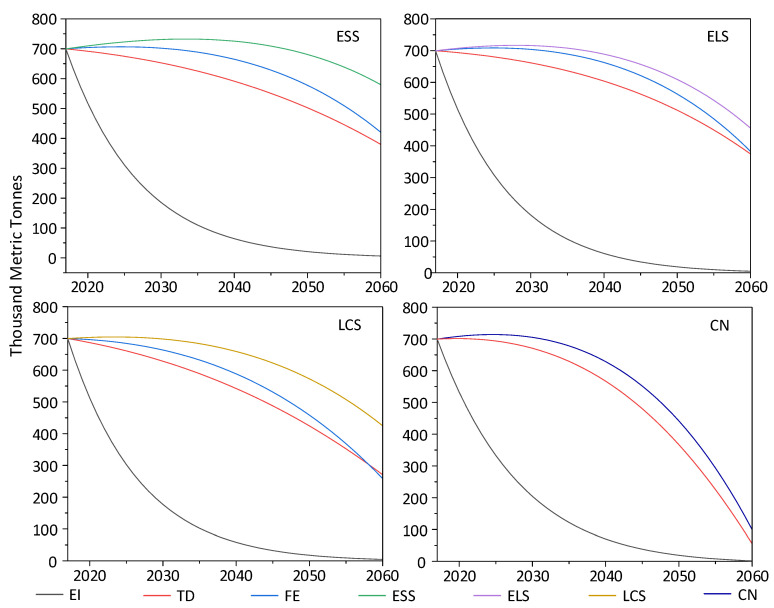
Sensitivity analysis results of the transportation sector under four scenarios (Unit: TMt).

According to the discussion of CO_2_ emissions of different perspectives in Section 3.2, the three main influencing factors are energy intensity, public transportation development and the share of fossil fuels. In order to quantitatively analyze the driving degree and driving direction of each influencing factor to the peak value, this paper, respectively, establishes three sub-scenarios in ELS by using the control variable method. The three sub-scenarios include reduce energy intensity (EI), develop public transport (TD) and reduce the proportion of fossil energy (FE). Under the premise that other influencing factors remain unchanged, the share of each influencing factor is changed successively [11]. The results are shown in Figure 7.

As for energy intensity, in ESS-EI, ELS-EI, LCS -EI, CN-EI, when energy intensity is reduced by 10%, there is no peak year and CO_2_ emissions are on a dramatically downward trend. In the peak year under different scenarios, the peak value of CO_2_ emissions will be reduced by 597.0 TMt in 2033, 492.0 TMt in 2028, 330.7 TMt in 2025 and 492.0 TMt in 2023, respectively; it is 81.5%,68.66%, 46.93% and 68.7% lower than the peak value under four scenarios, respectively. As for public transportation development, in ESS-TD, ELS-TD and LCS-TD, when individual transportation is decreased by 10%, there is no peak year and CO_2_ emissions are also on a downward trend. In the peak year under different scenarios, the peak value of CO_2_ emissions will be reduced by 95.6 TMt, 47 TMt and 32.8 TMt; it is 13.1%, 6.56% and 4.7% lower than the peak value under three scenarios, respectively. In CN -TD, individual transportation is decreased by 10%. In this sub-scenario, 2020 is the peak year and the peak year is five years earlier than CN. The peak value of CO_2_ emissions is 701.2 TMt; it is 1.8% lower than the peak value under CN in 2023. As for the share of fossil fuels, in ESS-FE, when proportion of fossil fuels is reduced by 10%, the peak year is 2024 and the peak value of CO_2_ emissions is 706.6 TMt; it is 3.5% lower than the peak value under ESS in 2033. The peak year is 9 years earlier than in the ESS scenario. In ELS -FE, when the proportion of fossil fuels is reduced by 10%, the peak year is 2025 and the peak value of CO_2_ emissions is 708.5 TMt; it is 1.1% lower than the peak value under ESS in 2028. The peak year is 3 years earlier than in the ELS scenario. In LCS -FE, when the proportion of fossil fuels is reduced by 10%, the peak value of CO_2_ emissions will be reduced by 14.5 TMt; it is 2.1% lower than the peak value under LCS. In CN -FE, when the proportion of fossil fuels is reduced by 10%, the peak year is 2023, 9 years earlier than in the CN scenario. In 2023, the peak value of CO_2_ emissions will be reduced by 9.9 TMt; it is 1.4% lower than the peak value under CN.

To sum up, energy intensity plays the dominant role in decreasing CO_2_ emissions of the urban transportation sector under cold climatic conditions. In ESS, CO_2_ emissions can be reduced by 81.5% through decreasing energy intensity which is the largest reduction in CO_2_ in the four scenarios. Public transportation development is the second driving factor in the urban transportation sector under cold climatic conditions. It can reduce the CO_2_ emissions by about 10%. As for the share of fossil fuels, its effect on reducing CO_2_ emissions of the urban transportation sector is slight.

## 4. Discussion

The differences in peak times and values are obvious depending on the scenario setup. The proportion of clean fuels in the ESS has increased compared to that in the BAU, and energy-saving technologies and energy efficiency have been improved due to the implementation of energy-saving policies. Compared with the peak under BAU, the peak under ESS is 9.7 TMt lower and 2 years earlier, and the value of CO_2_ emissions in 2060 is 79.15% of the value of CO_2_ emissions in the peak year under this scenario. For ELS, the introduction of energy efficiency policies in the transportation sector is an enhancement of ESS. Therefore, the peak time under ELS is 7 years ahead of the peak time under BAU, and the peak value under ELS is reduced to 25.5 TMt. The value of CO_2_ emission in 2060 is 63.61% of that in the peak year. The peak time under LCS is 10 years earlier than the peak time under BAU. The peak value under LCS is reduced to 28.2 TMt, and the CO_2_ emission in 2060 is only 60.31% of that in the peak year. Compared with other scenarios, CN is the most ideal scenario. The peak time under CN is 12 years earlier than that under BAU, and the CO_2_ emission in 2060 is only 13.95% of that in the peak year. Cars and taxis contribute the most to CO_2_ emissions from the transportation sector in cold regions due to the use of car heating and snow tires. At present, due to the technical problems of fuel cells in low temperature conditions, it is not possible to fully spread electricity and clean energy to the transportation field in cold regions. If there is a major breakthrough in this area, there will be great potential to reduce CO_2_ emissions. As for buses, in addition to technological breakthroughs, it can also optimize the construction of bus stations to make more people choose buses under cold climate conditions. Energy intensity has the largest impact on CO_2_ emissions and promoting the growth of traffic volume can significantly improve energy efficiency. Thus, increasing traffic volumes through strengthening road transportation infrastructure construction and rational planning of the metro transit routes are still the main measures to reduce CO_2_ emissions from the transportation sector in cold regions in the short term.

Energy intensity is related to factors such as the structure of energy use, energy efficiency and national policies. Measures and policies, such as improving fuel quality, promoting new technology, improving traffic equipment and promoting alternative fuels can reduce the energy intensity of the urban transportation sector effectively. Energy intensity plays the dominant role in decreasing CO_2_ emissions of the urban transportation sector under cold climatic conditions. However, due to the enduring winter, with a certain amount of snow, the travel efficiency of residents in cold regions is seriously decreased. Urban traffic operating conditions in winter are worse than in summer. Traffic accidents are more frequent, which decrease road capacity and make it difficult to reduce energy intensity. Therefore, it is difficult to reduce energy intensity in cold regions, resulting that developing public transportation is more effective on CO_2_ emission mitigation. Developing public transportation plays an important role in reducing CO_2_ emissions which achieves energy consumption reduction meanwhile ensuring transport services, and it is an effective way to alleviate environmental pressure. It can be concluded that with the rapid development of the metro and the introduction of new energy buses, to a certain extent, the transportation structure has improved. Replacing fossil fuels with clean energy and realizing the promotion of new energy vehicles are potential opportunities for the transportation sector to achieve emission reduction. Although the emission reduction is slight, restructuring the energy mix plays a significant role in the overall reduction in CO_2_ emission in the transportation sector under cold climatic conditions. In particular, the influence of temperature and road traffic conditions should be considered when adopting new technologies. Increasing the investment in charging piles and looking for available fuel cell technology in cold regions can contribute to the realization of CO_2_ emission reduction targets in the urban transportation sector more effectively. However, due to the impact of the climate, it is difficult to achieve a complete replacement of electricity in cold regions if there is no significant technological change and breakthrough. The trend of high energy consumption and low efficiency of the transportation sector in cold regions will continue.

LEAP is widely adopted by thousands of organizations in nearly 190 countries worldwide in the field of regional contribution commitment and climate mitigation planning [14]. Benefiting from modularization design, the LEAP model user can place more emphasis on emissions through the manipulation of related factors and the selection of sectoral mitigation techniques. CO_2_ emissions are measured by the internal accounting procedures of the LEAP model, so a result based on the assessment of the LEAP model will be more widely recognized internationally than other bottom-up models. The LEAP method can not only identify the main driving factors, but also clearly quantify the impacts of the driving factors on emission changes. Therefore, the method adopted can perform well when dealing with the issues that this study targets. From a method perspective, this study establishes an integrative method framework that allows us to explore the trend of the CO_2_ emission as well as quantify the driving effects of CO_2_ emissions from certain sectors under different scenarios; from a content perspective, this study focuses on the CO_2_ emissions from the urban transportation sector under cold climatic conditions that explores the discrepancy in CO_2_ emissions reduction pathways caused by climate heterogeneity. This study provides a reference for the work exploring the differences in CO_2_ emissions and mitigation pathways caused by climate heterogeneity from other economic sectors (such as agriculture sector, manufacture sector and building sector) or socioeconomic activities (household consumption).

Cooperative and differentiated emission reduction measures are needed to achieve the reduction in CO_2_ from the urban transportation sector under cold climatic conditions. The following policy implications are unraveled: (1) Due to the difficulties of decreasing the energy intensity, the government should give full consideration to the development of public transportation and promote the selection of public transportation by offering a more humanized service. In the current situation of increasing motor vehicle ownership, it is necessary to actively promote the replacement of existing energy sources with electricity and other clean energy and accelerate the construction of supporting facilities, charging piles and gas stations; (2) Winter transfer behavior in cold regions leads to bus passengers staying outdoors in cold for a long time, which reduces the comfort of bus travel. The introduction of an intelligent transportation system could improve the operation efficiency of the urban transportation department. Public transport authorities should increase the frequency of bus departures in winter, especially during morning and evening rush hours, to reduce the passengers’ waiting time; (3) The developing of new public traffic ways such as peak buses, customized buses and intelligent buses could reduce the growth rate of private cars to a certain extent.

## 5. Conclusions

This paper combines different modes of transportation and the proportions of various terminal energy sources in the transportation sector, and uses the LEAP model to set five different scenarios to predict the peak CO_2_ emissions of the transportation sector in cold regions. This paper also quantifies the driving degrees of influencing factors, explores pathways to reach the peak of CO_2_ emissions by 2030, and proposes feasible recommendations for the transportation sector in cold regions. The research indicates:

(1) The peak value and peak time of different scenarios are diverse. Under the five scenarios, the peak value is 704.7–742.1 TMt, and the peak year is during 2023–2035. Except for BAU and ESS, the other three scenarios can all achieve the peak of CO_2_ emissions before 2030.
(2) ELS is the optimal scenario to coordinate economic benefits and ecological environmental protection, with a peak time of 2028 and a peak value of 716.6 Mt. The peak time under ELS is 7 years earlier than that under BAU, and peak value is 25.5 TMt less than that under BAU. CO_2_ emissions in 2060 will be 63.61% of the peak year.
(3) This paper analyzes the CO_2_ emissions of different travel modes and different energy types, and finds that energy intensity is the primary driving factor for reducing CO_2_ emissions in the transportation sector in cold regions. If energy intensity is reduced by 10%, the peak value will be reduced by 492 Mt, accounting for 68.66% of the total CO_2_ emissions.
(4) However, due to the influence of a severe cold climate in cold regions, energy intensity is difficult to significantly reduce. Therefore, the development of transportation in cold regions is a more feasible factor to mitigate CO_2_ emissions. Sensitivity analysis states that the share of individual traffic is reduced by 10%, and the peak value is reduced by 47 TMt, accounting for 6.56% of total CO_2_ emissions.

In summary, it is recommended to promote the development of public transportation in cold regions by building a three-dimensional pedestrian system, building closed or semi-enclosed platforms and increasing the frequency of public transportation to reduce the CO_2_ emissions of the transportation sector. At the same time, it is necessary to accelerate the breakthrough of technical barriers and promote the substitution process of electric and hydrogen energy for traditional fuels, which will have a huge impact on the CO_2_ emission reduction in the transportation industry.

Due to time and conditions constraints, this study does not analyze the economic costs of each scenario. In future studies, the cost simulation calculation module in the LEAP software can be used for cost analysis to provide the government with a theory for establishing energy conservation and environmental protection incentives.

## Data Availability

Not applicable.
